# ADAM Metalloproteinase Domain 17 Regulates Cholestasis-Associated Liver Injury and Sickness Behavior Development in Mice

**DOI:** 10.3389/fimmu.2021.779119

**Published:** 2022-01-13

**Authors:** Wagdi Almishri, Liam A. Swain, Charlotte D’Mello, Tyson S. Le, Stefan J. Urbanski, Henry H. Nguyen

**Affiliations:** ^1^ Department of Microbiology, Immunology, and Infectious Diseases, Snyder Institute for Chronic Diseases, Cumming School of Medicine, University of Calgary, Calgary, AB, Canada; ^2^ Snyder Institute for Chronic Diseases, Cumming School of Medicine, University of Calgary, Calgary, AB, Canada; ^3^ Department of Pathology & Laboratory Medicine, Snyder Institute for Chronic Diseases, Cumming School of Medicine, University of Calgary, Calgary, AB, Canada; ^4^ Division of Gastroenterology and Hepatology, Snyder Institute for Chronic Diseases, Cumming School of Medicine, University of Calgary, Calgary, AB, Canada

**Keywords:** ADAM17 (a disintegrin and metalloprotease 17), TACE (TNF-α converting enzyme), cholestasis, autoimmune liver disease, inflammation, sickness behavior, therapeutic

## Abstract

Disintegrin and metalloproteinase domain-containing protein 17 (ADAM17) is a ubiquitously expressed membrane-bound enzyme that mediates shedding of a wide variety of important regulators in inflammation including cytokines and adhesion molecules. Hepatic expression of numerous cytokines and adhesion molecules are increased in cholestatic liver diseases including primary biliary cholangitis (PBC) and primary sclerosing cholangitis (PSC), however, the pathophysiological role of ADAM17 in regulating these conditions remains unknown. Therefore, we evaluated the role of ADAM17 in a mouse model of cholestatic liver injury due to bile duct ligation (BDL). We found that BDL enhanced hepatic ADAM17 protein expression, paralleled by increased ADAM17 bioactivity. Moreover, inhibition of ADAM17 bioactivity with the specific inhibitor DPC 333 significantly improved both biochemical and histological evidence of liver damage in BDL mice. Patients with cholestatic liver disease commonly experience adverse behavioral symptoms, termed sickness behaviors. Similarly, BDL in mice induces reproducible sickness behavior development, driven by the upregulated expression of cytokines and adhesion molecules that are in turn regulated by ADAM17 activity. Indeed, inhibition of ADAM17 activity significantly ameliorated BDL-associated sickness behavior development. In translational studies, we evaluated changes in ADAM17 protein expression in liver biopsies obtained from patients with PBC and PSC, compared to normal control livers. PSC and PBC patients demonstrated increased hepatic ADAM17 expression in hepatocytes, cholangiocytes and in association with liver-infiltrating immune cells compared to normal controls. In summary, cholestatic liver injury in mice and humans is associated with increased hepatic ADAM17 expression. Furthermore, inhibition of ADAM17 activity improves both cholestatic liver injury and associated sickness behavior development, suggesting that ADAM17 inhibition may represent a novel therapeutic approach for treating patients with PBC/PSC.

## Introduction

Disintegrin and metalloproteinase domain-containing protein 17 (ADAM17) is type 1 transmembrane metalloprotease with wide tissue expression in the body, that facilitates the cleavage and release of a variety of substrates involved in both the initiation and propagation of inflammation (1). In addition, ADAM17 is also expressed on the cell surface of various innate and adaptive immune cell populations (e.g. monocytes/macrophages, T lymphocytes, neutrophils) (2, 3). ADAM17 regulates the cleavage and release of over 80 different substrates including cytokines, cytokine receptors, growth factors, cell transport proteins, and various enzymes (1, 4). In the context of inflammation, ADAM17 cleaves and releases the cytokines IL-6 (and IL-6 receptor), tumor necrosis factor (TNF) α, and various adhesion molecules including ICAM-1 and VCAM-1, which critically regulate the development of numerous disease processes including sepsis, inflammatory bowel disease, psoriasis and multiple sclerosis (5–12). This broad role played by ADAM17 in the initiation and propagation of inflammation has made the development of ADAM17 inhibitors an appealing target for treating numerous chronic inflammatory and autoimmune conditions. Indeed, a number of ADAM17 inhibitors are in various stages of development for potential clinical use (1, 2, 13).

Enhanced ADAM17 activity has been shown to augment hepatic inflammation in animal models. Specifically, mice lacking TIMP3, the endogenous inhibitor of ADAM17, exhibit increased liver ADAM17 activity that is associated with increased proinflammatory cytokine production, hepatocyte necrosis, and hepatic leukocyte infiltration (14). In the setting of cholestatic liver disease, both progressive liver damage and impaired liver regeneration have been attributed, in part, to the differential hepatic expression of the proinflammatory cytokines TNF-α and IL-6; the processing and subsequent biological effects of which are mediated by ADAM17 (15–18). Increased circulating TNFα levels have been documented in both patients with cholestatic liver diseases, and in animal models of cholestasis (19–21). Polymorphisms in the TNFα gene, resulting in increased production of this cytokine, have been linked to the development of primary biliary cholangitis (PBC) (22). Increased hepatic TNFα levels have also been identified in patients with primary sclerosing cholangitis (PSC) (23). These findings suggest that ADAM17 activity, which critically regulates downstream soluble TNFα production, may be increased in cholestatic liver diseases. Interestingly, the hepatoprotective effects of ursodeoxycholic acid (UDCA), the keystone therapy in PBC, has been attributed in part to its inhibitory effect on mature ADAM17 formation (24).

PBC and PSC patients commonly experience a variety of comorbid psychological manifestations (i.e., fatigue, depression, anxiety, social withdrawal), also termed sickness behaviors, that lead to detriments in patient quality of life and an increased burden of disease (25–27). UDCA is the current mainstay of treatment for cholestatic liver diseases, including PBC. However, although it prevents disease progression in PBC patients, UDCA treatment does not typically improve disease-associated behavioral symptoms (28). This highlights an unmet medical need for new treatment approaches to beneficially impact these symptoms (collectively termed sickness behaviors). Interestingly, sickness behaviors experienced in cholestatic patients can be readily modelled in animal models of cholestatic liver disease including BDL mice (29, 30), and are characterized by increased immobility and a reduced propensity to engage in social interactions. Although the specific mechanisms leading to the development of sickness behaviors in cholestatic liver disease are not yet fully understood, alterations in pro-inflammatory cytokine production (including TNFα and IL-6), cellular adhesion molecule expression on cerebral endothelial cells, production of the chemokine monocyte chemoattractant protein-1 (MCP-1) by brain microglia, and cerebral recruitment of inflammatory monocytes have been broadly implicated (31–33). Moreover, a recent study showed that the ADAM17–mediated release of soluble TNFα enhanced endothelial cell expression of both adhesion molecules and MCP-1, with inhibition of ADAM17 preventing monocyte adhesion to endothelium (8). Current findings therefore suggest that ADAM17 may regulate the initiation/progression of liver injury in cholestatic liver disease, as well as the development of cholestatic liver disease-associated sickness behavior development.

The broad biological impact of liver disease-related increase in ADAM17 expression and activity could therefore make inhibition of ADAM17 activity a potential novel target for treating both cholestasis associated liver injury and sickness behavior development. Therefore, we evaluated the hepatic expression/activity of ADAM17, and the impact of ADAM17 inhibition, on liver injury and sickness behavior development in a well characterized murine model of cholestasis. To evaluate for potential applicability of our findings in the clinical setting, we characterized cholestatic liver disease-related changes in ADAM17 protein expression in liver biopsies obtained from patients with PBC and PSC.

## Methods and Materials

### Animal Model of Cholestatic Liver Disease Due to Bile Duct Ligation (BDL)

Male C57BL/6 mice 8-10 weeks of age (Jackson Labs, Bar Harbor, Maine) were housed in a light controlled room at 22°C with a 12-hour day/night cycle and free access to water and food. All animals were treated humanely under the University of Calgary Animal Care Committee guidelines, and all experiments were performed in accordance with the guidelines of the Canadian Council on Animal Care. The University of Calgary Animal Care Committee prospectively approved the animal research prior to study initiation. Cholestasis was induced *via* bile duct ligation and resection (BDL), which is a well characterized and widely used model of cholestatic liver injury (16, 34). Sham resection consisted of laparotomy with bile duct manipulation without ligation or resection. All surgeries were performed under isoflurane with efforts made to minimize animal suffering. Studies were performed at 10 days post-surgery, a time when mice are overtly cholestatic (34). Animals were sacrificed on day 10 using inhaled isoflurane and subsequent studies were carried out as outlined in the methodology section below. Necrotic areas within the liver of BDL vs sham mice were quantified using ImageJ software (35) in H&E stained liver sections, and the percentage of necrotic vs normal liver tissue area calculated using the formula: ([number of total pixels of necrotic areas/total number of pixels of entire liver section] x 100) (36). The severity of liver injury and degree of bile duct proliferation were also evaluated by a single expert liver histopathologist blinded to treatment groups (S.U.) (30). The degree of bile duct proliferation was assessed using a subjective scoring system by the blinded histopathologist (S.U.). A score of 0 (no bile duct proliferation) to 3 (marked bile duct proliferation) was utilized. In addition, serum alanine aminotransferase (ALT) and total bilirubin levels were measured using standard techniques (Calgary Laboratory Services, Calgary, Alberta).

### Determination of BDL-Induced Changes in Hepatic ADAM17 Protein Expression and Enzymatic Activity

(a) Hepatic ADAM17 protein expression: Liver samples from day 10 BDL and sham control mice were obtained as described above, placed in 5% formalin, and embedded in paraffin. Samples were sectioned 5 µm thick and antigen retrieval performed by incubating samples in 1X citric acid for 20 minutes at 80°C. Endogenous peroxidase was blocked with 3% hydrogen peroxide. Slides were then blocked with 4% goat serum. Rabbit monoclonal anti-ADAM17 antibody (10µg/mL; LifeSpan Biosciences; Seattle WA) was applied at 4°C overnight, followed by biotinylated goat anti-rabbit IgG (10µg/mL; 90 minutes room temperature) along with avidin biotin complex (Vectastain Elite ABC; Burlingame, CA) and Nova Red peroxidase substrate (Vector Labs; Burlingame CA). Positive ADAM17 protein expression was indicated by red staining.

(b) Hepatic ADAM17 enzymatic activity determination: ADAM17 activity was measured in liver homogenates obtained from sham and BDL mice using a commercially available fluorometric ADAM17 Activity Assay Kit (Anaspec, Freemont, CA) (37). Briefly, under inhalational anesthesia, mouse livers were flushed with cold Phosphate Buffered Saline (PBS), surgically removed, and weighed. Livers were then homogenized with assay buffer containing Triton-X 100 and incubated for 15 minutes at 4°C. Samples were then centrifuged at 2000xg and supernatants collected. 96-well plates were prepared as per the manufacturer’s protocol, including appropriate controls (sample alone, buffers alone, ADAM17 substrate alone, and 20 µM ADAM17 inhibition compound containing TAPI-O). A reference standard was also prepared on the same plate for ADAM17 activity quantification purposes. Endpoint fluorometric intensity readings were obtained one-hour post-incubation (Fluostar Optima; BMG Labtech, Durham, NC); with excitation and emission wavelengths at 490 nM and 520 nM, respectively. Fluorometric activity was normalized per mg of protein, and ADAM17 activity expressed as relative units reflecting only the proportion of ADAM17 activity inhibited by the specific ADAM17 inhibitor, TAPI-O.

### Isolation and Flow Cytometric Phenotyping of Hepatic Immune Cells From BDL and Sham Mice

Livers from cholestatic and control mice at day 10 were perfused with 20 mL of ice-cold PBS. Immune cell isolation was carried out as previously described (38, 39). In brief, mechanical dissection of whole livers was completed by passing tissue through a 70µm filter using the blunt end of a syringe with 10% RPMI + fetal bovine serum. Immune cells were isolated utilizing density gradient centrifugation overlaying 37% over top of 70% Percoll^®^. Immune cells were labelled with fluorescent tagged antibodies, and immunophenotyping of isolated hepatic immune cells expressing ADAM17 in BDL and sham mice assessed using multicolor flow cytometry. For cell surface staining, cell isolates were first treated with Fc receptor blocker (2.0 µg/tube; CD16/CD32; 93; eBioscience, San Diego, CA) prior to staining. Predominant immune cell types observed in our human samples guided immunophenotyping in cholestatic mice. Hepatic T lymphocytes were identified using anti-mouse CD3e (0.25 µg/tube;145-2C11; eBioscience, San Diego, CA), and monocytes/macrophages identified by co-labelling cells with anti-mouse CD11b (0.125 µg/tube; M1/70; eBioscience, San Diego, CA) and LY6C (0.25 µg/tube; HK1.4; eBioscience, San Diego, CA). ADAM17 expression was determined using anti-mouse ADAM17 Alexa Fluor 647 (0.5 µg/tube; ab150115; Abcam, Toronto, Canada). Cell populations of interest were acquired using the Attune™ Acoustic Focusing Cytometer (Applied Biosystems; Burlington ON). Data was analyzed using FlowJo^®^ software (Treestar, Ashland OR). Gating was carried out as follows: Live cells were gated, and duplet cells were excluded. Using forward scatter (FSC) and side scatter (SSC) regions coinciding to cells of interest were included. Fluorescence-minus-one (FMO) controls were used for the accurate designation of cells with fluorescence above background levels (38). Appropriate isotype controls were used to determine the specificity of all antibodies used for flow cytometry. Cell numbers were calculated based on the percentage of cells found in the gate of interest and the total cell numbers isolated from each liver.

### Impact of ADAM17 Inhibition on Liver Injury and Sickness Behavior Development in Cholestatic Mice

(i) Impact of ADAM17 inhibition on BDL-associated liver damage: Selective ADAM17 inhibitors have been developed for possible clinical application (2, 40). DPC 333 is a well characterized selective ADAM17 inhibitor, with potent oral and parenteral bioactivity in rodents and humans (also called BMS 561392; kind gift of Bristol-Myers-Squibb Canada) (13). We confirmed the effectiveness of ADAM17 inhibition by DPC 333 in preliminary pilot studies. To do this, mice were treated with DPC 333 (10 mg/kg, ip) or vehicle (n=2 mice/group) 30 minutes prior to ip endotoxin administration (1 mg/kg; O111:B4; Sigma), and 2 hours later serum TNFα levels were measured using a Luminex^®^ assay (Eve Technologies, Calgary, Canada). DPC 333 reduced endotoxin-induced elevations in serum TNFα levels by greater than 50% (data not shown). Therefore, in subsequent studies, BDL mice were treated with either DPC 333 (10 mg/kg ip twice daily; dose previously shown to effectively inhibit ADAM17 activity in mice) (13) or saline vehicle, starting at day 1 post-BDL surgery and continuing until day 10 post-BDL surgery, at which time mice were sacrificed. Livers were then removed for H&E staining (for histological quantification of liver damage) and blood samples taken for biochemical assessment of liver injury (i.e. serum ALT and total bilirubin levels). Hepatic necrotic areas were quantified using Image J software as described above. Evaluations were also performed by an expert, blinded liver histopathologist (S.U).

(ii) Effects of ADAM17 inhibition on BDL-associated sickness behavior development: Sickness behavior development in mice was assessed using the social exploration/investigation paradigm. Using this paradigm, we have previously shown that BDL mice reproducibly develop sickness behaviors which are readily quantified (34, 41). Briefly, prior to testing, test mice were housed in individual cages for a period of 24 hours. Experiments were performed between 7:00 and 10:00 am on Day 10 post-surgery. A 3-4 week old juvenile male C57BL/6 mouse was introduced into the home cage of the test mouse, and social investigative behavior assessed for a period of 10 minutes by two blinded observers (30, 34, 41). Two aspects of social exploratory behavior were quantified: (i) total time the test mouse spent engaging in social investigative behavior, and (ii) the total time the test mouse remained immobile after the introduction of the juvenile mouse into the test cage. DPC 333 or vehicle treated BDL mice were assessed on day 10 post-surgery for the development of sickness behaviors using this social investigation paradigm.

### Cholestatic Liver Disease-Associated Alterations in Hepatic ADAM17 Protein Expression in PBC and PSC Patients

Hepatic ADAM17 protein expression was evaluated in percutaneous liver biopsy samples obtained from patients (University of Calgary ethics protocol number E-23683) with the cholestatic liver diseases primary biliary cholangitis [PBC; n=3] and primary sclerosing cholangitis [PSC; n=3]. PBC/PSC liver sections were compared to biopsies from normal liver (n=3). Patients had all been assessed by a hepatologist within the University of Calgary Liver Unit, and other aetiologies of chronic liver diseases ruled out. The diagnosis of PBC and PSC were all confirmed with liver biopsies in these patients. Antigen retrieval was performed using 1 X citric acid at 80°C for 20 minutes. Primary staining was done using a monoclonal rabbit anti-ADAM17 antibody (10µg/mL overnight at 4°C; LifeSpan Biosciences; Seattle WA), followed by goat anti-rabbit IgG along with Avidin Biotin Complex and Nova Red peroxidase substrate staining (Vector Labs, Burlingame CA). Sections were counter-stained with Hematoxylin. Appropriate staining controls were employed to ensure antibody specificity.

### Immune Cell ADAM17 Expression in Hepatic Inflammatory Cell Infiltrates in PBC and PSC Patients

Immunostaining of PSC/PBC patient liver biopsy samples for T lymphocytes used anti-CD3 (1:100 dilution; Thermo Fisher) as a pan-T cell marker, using standardized protocols (Calgary Laboratory Services, Calgary, Canada). Immune cell ADAM17 expression was determined as follows: Antigen retrieval was performed using 1X citric acid at 80°C for 20 minutes. Slides were blocked with 4% goat and 4% donkey serum in PBS. Primary staining was done using a monoclonal rabbit anti-ADAM17 antibody (10µg/mL; LifeSpan Biosciences; Seattle WA) in combination with (i) T cell subset markers, identified with mouse anti-human CD8a (AMC908; eBioscience; San Diego, CA) or mouse anti-human CD4 (N1UG0; eBioscience; San Diego, CA), or (ii) monocytes/macrophages, identified with mouse anti-human CD68 (815CU17; eBioscience; San Diego, CA) at 4°C overnight. Secondary antibody staining was done using both goat anti-rabbit IgG Alexa Fluor 488 and Donkey anti-mouse IgG Alexa Fluor 647 (90 minutes at room temperature). Nuclei were stained using DAPI, as per manufacturer protocol (ThermoFisher Scientific; Eugene, OR). Liver immunofluorescent microscopy was performed using a multiphoton confocal microscope (Nikon A1 MP+ multiphoton confocal microscope; Melville NY). Excitation laser wavelengths of 402.5 nm and 639.7 nm were used for the identification of ADAM17 and immune cell markers (CD4/CD8/CD68), respectively.

### Statistical Analysis

Results are expressed as mean ± standard error of the mean (SEM). Data were analyzed using GraphPad Instat software. For comparison between two means, Student’s unpaired *t*-test was used. For multiple comparisons, one-way ANOVA followed by Student-Neuman-Keuls *post hoc* test was used. p < 0.05 was considered significant.

## Results

### Hepatic ADAM17 Activity and Expression Is Increased in BDL Mice

**Figure 1:**
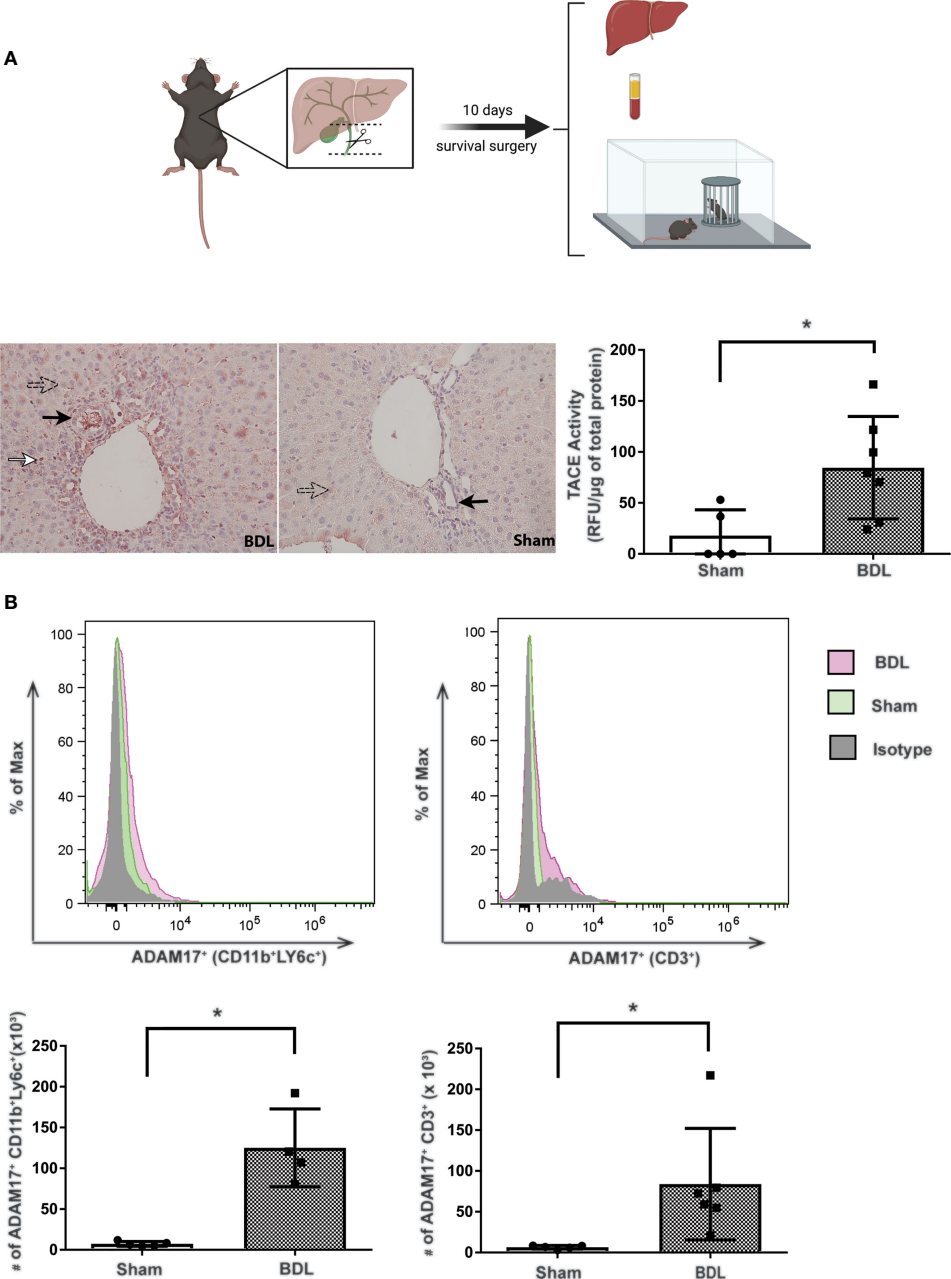
Increased hepatic ADAM17 activity and protein expression in cholestatic mice. Diagram of experimental design. Bile duct ligation (dotted line) with resection in between ligatures was performed in BDL mice. Mice were overtly cholestatic 10 days post surgery at which point liver histology, liver immune cell isolation, liver TACE activity, serum biochemistry and behavioral studies were performed. **(A)** Qualitative hepatic ADAM17 protein expression in liver tissue from day 10 BDL and sham mice. Representative images illustrating positive ADAM17 staining (red colour) being mainly localized to hepatocytes (perforated arrows), cholangiocytes (black arrows), and immune cells within inflammatory infiltrates (white arrows). ADAM17 protein expression was also detected in sham liver, however the increased ADAM17 expression was more readily observed in BDL livers. Images acquired at 20X and are representative of similar findings in n = 7 BDL and n = 5 sham mice. Quantitative hepatic ADAM17 enzymatic activity (expressed as Relative Fluorescent Units/μg of total liver protein). The mean ± SEM was from n = 7 BDL and n = 5 Sham mice; *p < 0.05. These experiments were repeated twice with similar results. **(B)** Increased ADAM17 expression in hepatic tissue derived immune cells. Flow cytometry histogram and absolute cell count of ADAM17+ expression in monocytes and T lymphocytes isolated from day 10 BDL and sham livers. Absolute cell counts are represented as mean ± SEM for data from n = 4-6 BDL and n = 4-5 sham mice; *p < 0.05). This experiment was repeated twice with similar results.

Consistent with previous findings, BDL mice developed overt liver injury and cholestasis as reflected by significant increases in serum bilirubin and ALT levels vs sham control mice (ALT: sham 12.1 ± 2.2 U/L vs BDL 754.0 ± 77.8 U/L; p<0.02; total bilirubin: sham 9.2 ± 8.2 µmol/L vs BDL 262.0 ± 29.6 µmol/L; p<0.05; n=4-6 mice/group). ADAM17 protein expression was increased in liver tissue from BDL vs sham mice as seen on immunohistochemistry (**Figure 1A**). Consistent with this observation, ADAM17 enzymatic activity was also significantly increased in liver homogenates in BDL vs sham mice (**Figure 1A**). Increased hepatic ADAM17 protein expression was identified in hepatocytes, cholangiocytes and immune cells which had infiltrated the liver (**Figure 1A**). To further characterize the ADAM17 expressing infiltrating immune cells, hepatic immune cells were isolated and evaluated with flow cytometry. A significant increase in the total numbers of ADAM17 expressing monocytes and T lymphocytes were documented in BDL vs sham mice (**Figure 1B**). No differences in mean fluorescence intensity (MFI) for ADAM17 expression were found in any of the immune cell populations in either experimental group (data not shown).

### *In Vivo* Inhibition of ADAM17 Activity With DPC 333 Leads to an Improvement in Liver Biochemistry, Histological Liver Damage, and Attenuates Sickness Behavior Development in BDL Mice

**Figure 2:**
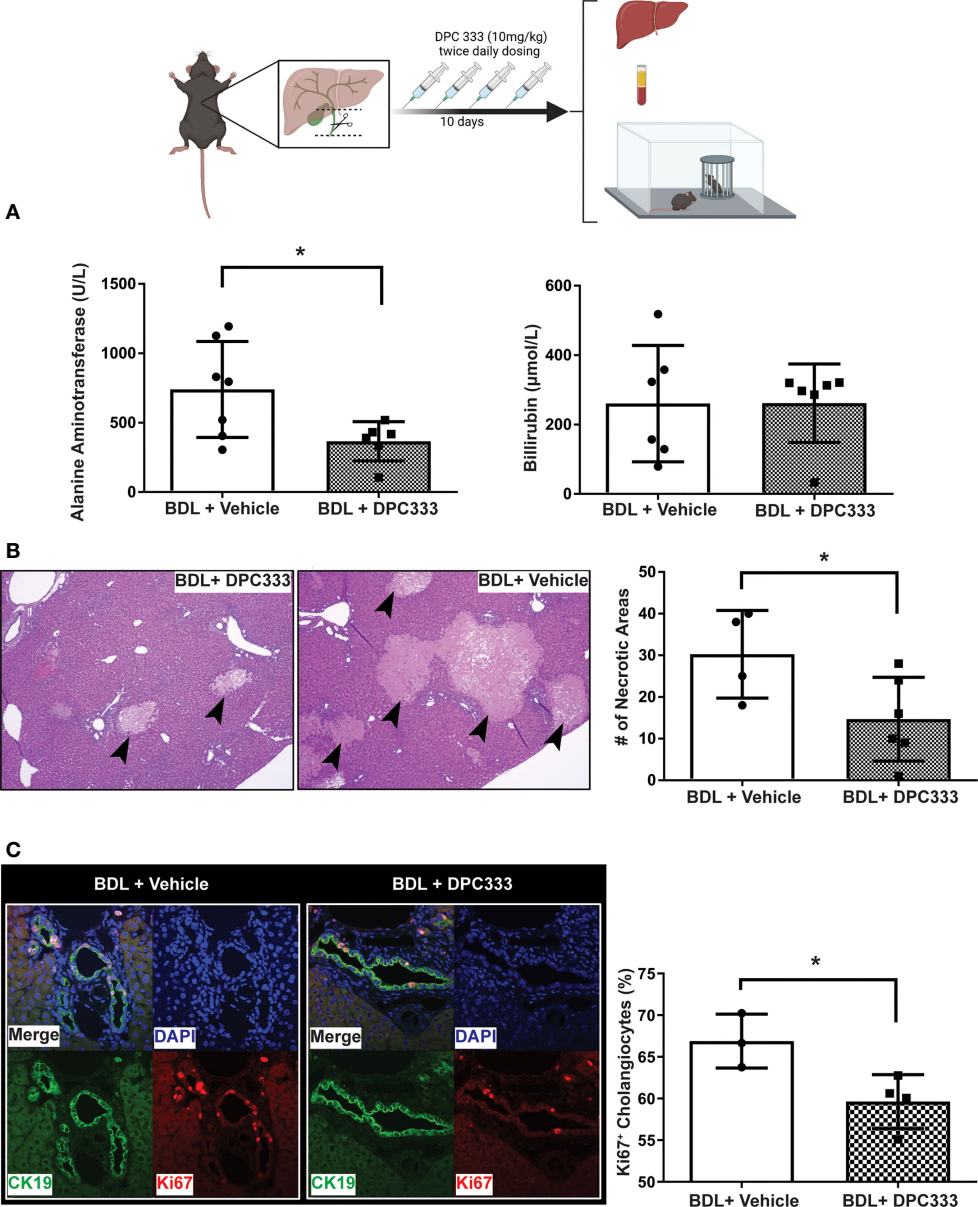
Inhibition of ADAM17 enzymatic activity attenuates liver injury in BDL mice. Diagram of experimental design depicting bile duct ligation (dotted line) with resection in between ligatures in BDL mice. Twice daily treatment with DPC 333 (10 mg/kg) was provided *via* intraperitoneal injection. Mice were overtly cholestatic 10 days post surgery at which point experimentation was completed. **(A)** Improvement in serum ALT levels (but not total bilirubin levels) in DPC 333-treated BDL vs vehicle-treated BDL mice (mean ± SEM of data from n = 7 DPC 333 treated BDL mice and n = 7 vehicle treated BDL mice; *p < 0.05). **(B)** Representative H&E stained liver sections showing reduction in areas of liver cell necrosis (thick black arrows) in DPC 333-treated vs vehicle-treated BDL mice. Images are 20X and are representative of n = 7 DPC 333 treated group and n = 6 vehicle treated group). Bar graph shows counting of necrotic areas by blinded liver pathologist (DPC 333 treated 14.7 ± 4.1 vs. vehicle treated 30.2 ± 5.3; p < 0.05; n = 6 BDL + DPC 333 mice and n = 4 BDL + vehicle mice). **(C)** Attenuation of bile duct proliferation in BDL mice with DPC 333 treatment vs vehicle (PBS). Representative immunohistochemistry of BDL livers with and without DPC 333 showing CK19 (green), Ki67 (red) and nuclear staining (blue). Bar graph quantification done in a blinded manner with significant reduction in Ki67+ cholangiocytes in BDL mice treated with DPC 333 vs vehicle (PBS). *p < 0.033; n = 3 and 4 mice/group.

**Figure 3:**
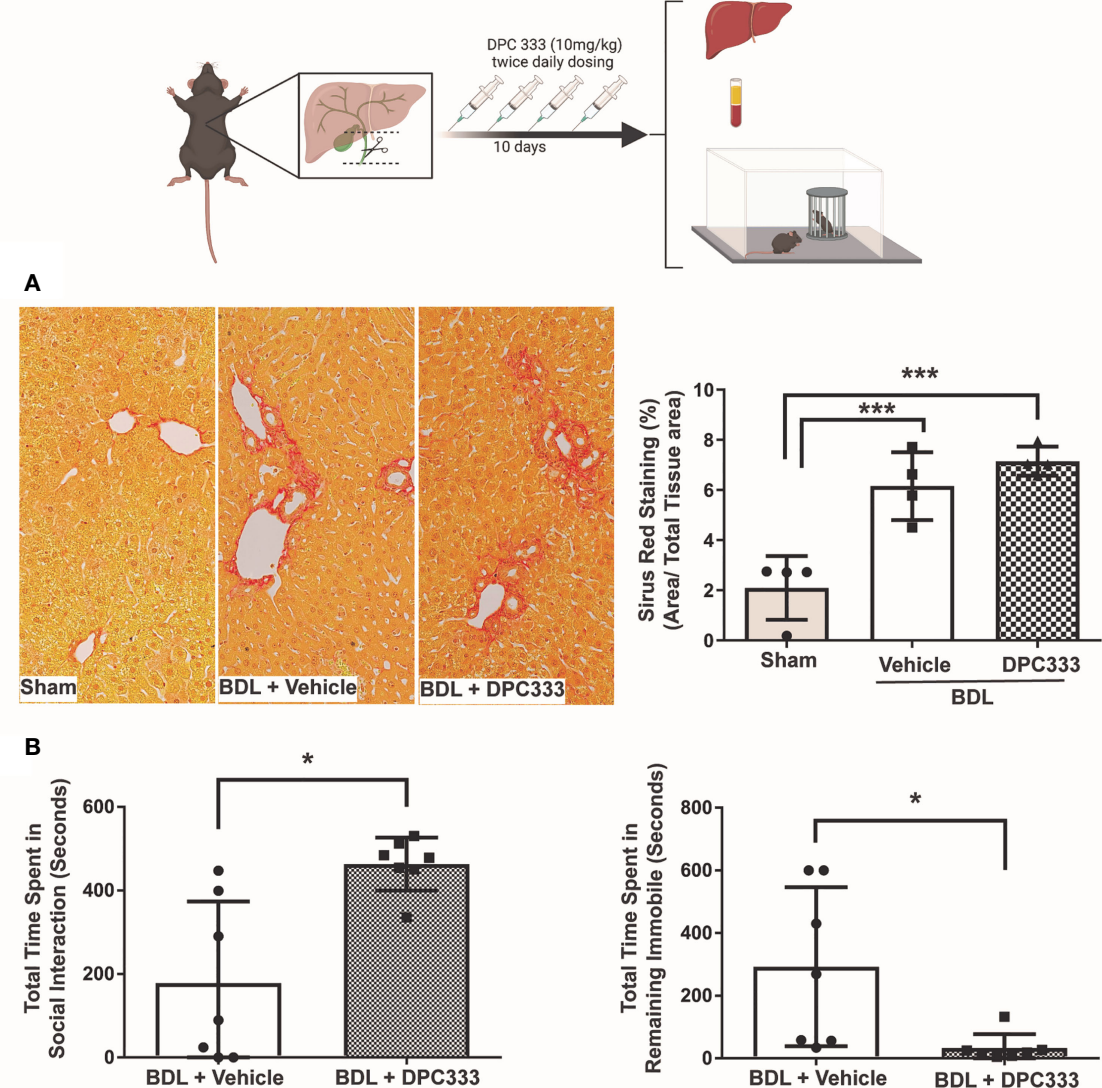
Inhibition of ADAM17 enzymatic activity attenuates sickness behavior development in BDL mice. Diagram of experimental design depicting bile duct ligation (dotted line) with resection in between ligatures in BDL mice. Twice daily treatment with DPC 333 (10 mg/kg) was provided *via* intraperitoneal injection. Mice were overtly cholestatic 10 days post surgery at which point experimentation was completed. **(A)** DPC 333 treated BDL mice did not result in a significant reduction in liver fibrosis as shown by Sirius red staining and blinded quantification. **(B)** Improvement in BDL-associated sickness behaviors in day 10 DPC 333-treated vs vehicle-treated BDL mice as reflected by a significant increase in the time DPC 333-treated BDL mice spent engaging in social investigative behavior vs vehicle-treated BDL mice, and a significant reduction in the time DPC 333-treated BDL mice spent remaining immobile compared to vehicle-treated BDL mice. *p ≤ 0.05. Graphs show the mean ± SEM of n = 7 DPC 333-treated BDL mice, and n = 7 vehicle-treated BDL mice. All experiments were repeated twice with similar results. ***p < or equal to 0.001.

(i) Reduction of liver injury in BDL mice with ADAM17 inhibition: Inhibition of ADAM17 activity with DPC 333 administration resulted in a significant decrease in serum ALT levels in BDL mice (**Figure 2A**), but not serum bilirubin levels (**Figure 2A**), compared to the vehicle-treated BDL group. Paralleling improvements in serum ALT levels, DPC 333 treatment in BDL mice significantly attenuated liver damage, shown histologically as a significant reduction in hepatic necrosis (**Figure 2B**). Quantification of total liver necrotic area (expressed as % necrotic area) demonstrated a significant reduction in hepatic necrosis in DPC 333 treated BDL mice, vs vehicle treated BDL mice (3.0 ± 1.0% vs 11.0 ± 2.2% respectively; p< 0.05; n=7 mice in BDL+DPC 333 group and n=6 mice in BDL + vehicle group). A significant reduction in cholangiocyte proliferation (CK19^+^Ki67^+^ cells), a histological feature commonly encountered in cholestatic liver injury (**Figure 2C**), was also documented in DPC 333-treated BDL mice. The findings of reduction in liver injury severity with DPC 333 treatment was also confirmed by our liver histopathologist (S.U) blinded to treatment group who counted total number of hepatic necrotic areas per liver section in DPC 333 treated vs vehicle treated BDL mice: (DPC 333 treated 14.7 ± 4.1 vs. vehicle treated 30.2 ± 5.3; p<0.05; n=6 BDL + DPC 333 mice and n=4 BDL + vehicle mice) (**Figure 2B**). In addition, DPC 333 treatment also resulted in a reduction in a subjective score of bile duct proliferation in DPC 333 treated vs vehicle treated BDL mice, as estimated by our blinded histopathologist (Score from 0 to 3: Score = 1.3 ± 0.2 for DPC 333 treated and Score = 2.3 ± 0.3 for vehicle treated; p<0.05; n=6 BDL+DPC 333 mice and n=4 in BDL + vehicle mice). The impact of ADAM17 inhibition on liver fibrosis development in response to BDL surgery was assessed *via* Sirius red staining of liver sections to detect collagen deposition. BDL mice showed increased Sirius red staining compared to sham controls. However, ADAM17 inhibition in BDL mice by DPC 333 treatment did not alter the development of hepatic fibrosis (**Figure 3A**).

(ii) ADAM17 inhibition attenuates sickness behavior development in BDL mice: BDL mice develop overt sickness behaviors, as reflected by a decrease in time spent engaging in social investigation and an increase in time remaining immobile (30, 34, 41). ADAM17 inhibition with DPC 333 treatment resulted in a significant reduction in sickness behavior development in BDL mice compared to vehicle-treated BDL mice, manifested as a significant increase in the time DPC 333-treated BDL mice spent engaging in social investigating behavior, and a decrease in time they spent remaining immobile, compared to vehicle treated BDL mice (**Figure 3B**).

### Enhanced Hepatic ADAM17 Protein Expression in the Cholestatic Liver Diseases PBC and PSC

**Figure 4:**
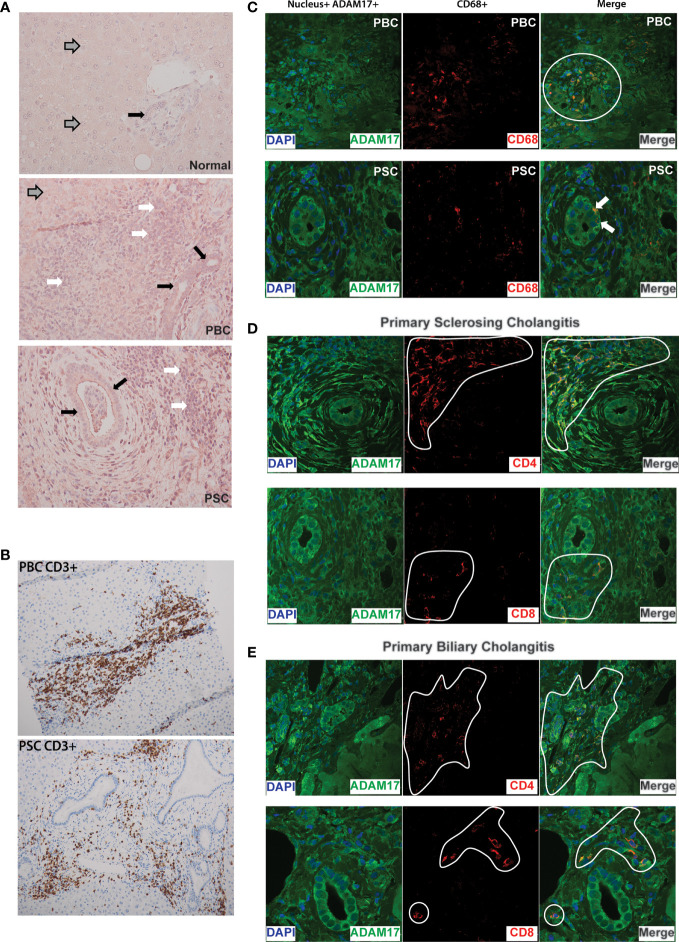
**(A)** Increased ADAM17 protein expression in PBC and PSC livers: Representative immunohistochemical images (20X) showing increased hepatic ADAM17 protein expression in patients with Primary Biliary Cholangitis (PBC; middle panel) and Primary Sclerosing Cholangitis (PSC; bottom panel) compared to normal liver (top panel). Images are representative of similar findings in n = 3 liver samples/group. For all three panels grey arrows indicate hepatocytes, white arrows indicate inflammatory infiltrate, and solid black arrows indicate cholangiocytes. Relative to normal liver, ADAM17 protein expression (red staining) is markedly increased in PBC and PSC. **(B)** Portal inflammatory infiltrates in PBC and PSC are mainly composed of T lymphocytes: CD3+ T cells within immune cell infiltrates in inflamed portal areas of liver biopsies from patients with PBC (Top Panel) and PSC (Bottom Panel). Images acquired at 20X and are representative of similar findings in n = 3 patients/group. Positive staining is indicated by amber/brown color. **(C)** Expression of ADAM17 (green colour) on the cell surface of CD68+ monocytes/macrophages (red colour) in PBC and PSC liver samples. Nuclei are stained blue. Images are representative of similar findings in n = 3 patients/group. ADAM17+ CD68+ cells were readily identified within inflammatory cell infiltrates within portal areas (white circle in PBC sample). Interestingly, in PSC liver sections only, ADAM17+CD68+ monocytes could also be identified infiltrating between bile duct (BD) cholangiocytes (denoted by white arrow). **(D)** ADAM17 (green colour) expression on CD4^+^ and CD8^+^ T lymphocyte subsets (red colour) in liver biopsies from patients with PSC. Nuclei are stained blue. Images are representative of similar findings in n = 3 PSC patients. ADAM17 expression by both CD4^+^ and CD8^+^ T cells was readily noted within inflammatory infiltrates (denoted by white highlight) within expanded portal areas in PSC livers. Increased ADAM17 expression was also noted in hepatocytes (not shown) and biliary epithelium/cholangiocytes. **(E)** ADAM17 (green colour) expression on CD4^+^ (top panels) and CD8^+^ (bottom panels) T lymphocyte subsets (red colour) in liver biopsies from patients with PBC. Nuclei are stained blue. Images are representative of similar findings in n = 3 PBC patients. ADAM17 expression by both CD4^+^ and CD8^+^ T cells was readily identified within inflammatory infiltrates (denoted white line) within expanded portal areas in PBC livers. Increased ADAM17 expression was also noted in hepatocytes and biliary epithelium/cholangiocytes. All images are representative of other patient PBC and PSC samples. Experiments were repeated total 2 times with similar results.

Given the findings of ADAM17 inhibition attenuating liver injury and sickness behavior in BDL mice, we were interested in evaluating ADAM17 in patients diagnosed with chronic autoimmune cholestatic liver disease. We noted ADAM17 protein expression was strikingly increased in liver biopsy samples obtained from patients with both PBC and PSC compared to normal controls (**Figure 4**). In both PBC and PSC livers, ADAM17 protein expression (red stain) was readily noted in hepatocytes (most notably in areas of the liver adjacent to active inflammation), and in areas of hepatic immune cell infiltration. When immune cell rich areas within the livers of PBC and PSC patient were characterized by immunohistochemistry, T lymphocytes (CD3^+^) were found to be the predominant cell type present (**Figure 4B**). To address this in more detail we next evaluated the different hepatic immune cell populations of PBC and PSC patients for ADAM17 expression; specifically CD4^+^ T cells, CD8^+^ T cells, and monocytes/macrophages (CD68^+^ cells) which are known to express ADAM17 (42, 43). In normal liver samples there was no portal area inflammatory cell infiltration evident (data not shown). In PBC and PSC livers, ADAM17 expressing CD68^+^ (i.e., monocyte/macrophage) immune cells were noted in hepatic portal inflammatory cell infiltrates (PBC; **Figure 4C**; top row, and PSC; **Figure 4C**; bottom row associated with classic concentrically fibrosed duct). However, ADAM17 expressing CD68^+^ cells were less abundant than were ADAM17 expressing T-cells in inflamed portal areas in PBC and PSC livers. CD68 positive staining immune cells were also observed within hepatic sinusoids, and these cells were morphologically consistent with Kupffer cells (not shown). Evaluation of ADAM17 expressing T cells in liver sections from both PBC (**Figure 4E**) and PSC (**Figure 4D**) patients demonstrated that portal T cell inflammatory infiltrates were significantly enriched in both CD4^+^ and CD8^+^ ADAM17-expressing T cell subsets. Moreover, ADAM17^+^ T cells were also observed directly infiltrating the biliary epithelium (**Figure 4E**).

## Discussion

The role of ADAM17 in regulating inflammation relates to its ability to process various substrates, including proinflammatory cytokines (e.g. TNFα), cytokine receptors (e.g. TNFRI/TNFRII, IL-6Rα), adhesion molecules (L-selectin, VCAM-1, ICAM-1) and growth factors (e.g. EGFR ligands) (2–4, 40). Increased tissue ADAM17 expression has been reported in different immune-mediated inflammatory diseases, including rheumatoid arthritis, psoriasis and inflammatory bowel disease highlighting its role in mediating downstream injury/inflammation (6–8). This has made ADAM17 inhibition an attractive and potential upstream therapeutic target for these various conditions (2, 4, 40, 44, 45). Although a number of studies have highlighted ADAM17 activity as an important regulator of liver injury and repair (14, 46–48), the role of ADAM17 in the context of cholestatic liver disease is poorly understood. We have identified enhanced hepatic expression and enzymatic activity of ADAM17 in a mouse model of cholestatic liver injury. Moreover, cholestatic liver injury severity in this model as reflected biochemically by serum ALT levels and histopathologically by liver damage scores and bile duct proliferation (49), was attenuated by treatment with the specific ADAM17 inhibitor DPC 333. Of note, DPC 333 treatment did not impact the BDL-associated increase in serum bilirubin levels, which is not surprising given that in this model the common bile duct is surgically completely blocked. Interestingly, ADAM17 inhibition did not attenuate liver fibrosis development in BDL mice, possibly reflected by the relatively modest fibrotic response in day 10 BDL mice. Therefore, murine models employing hepatoxic agents such as dimethylnitrosamine, diethylnitrosamine, and carbon tetrachloride over a longer duration of time may be more appropriate to study the role of ADAM17 in liver fibrosis and cirrhosis development (50).

Our group has previously defined critical roles for the ADAM17-regulated cytokines TNFα and IL-6, cytokine receptor TNFRI, and immune cell adhesion molecule VCAM-1, in cholestasis associated sickness behavior development in BDL mice (29, 34, 41, 51). Additionally, our group (30, 52) and others (53) have previously shown that the BDL model is associated with elevated circulating serum IL-6 and TNFα levels along with the reproducible development of liver injury and associated sickness behaviors. Interestingly, both serum IL-6 and TNF-α levels have been reported to be linked with PBC and PSC disease process (54–56). In our current study, we now show that inhibition of ADAM17 ameliorates cholestasis-associated sickness behavior development, as reflected by an increase in time spent engaging in social interaction and a decrease in time spent remaining immobile in DPC 333-treated cholestatic mice. Patients with cholestatic liver disease (e.g. PBC and PSC) commonly experience a number of extrahepatic symptoms including fatigue, malaise and loss of social interest. These symptoms have been collectively called sickness behaviors, and by definition must originate by changes occurring within the brain (29, 57). Importantly, these symptoms often represent a significant societal and personal burden for cholestatic patients, and are strongly associated with detriments in patient health-related quality of life (57, 58). However, symptom severity in cholestatic patients correlates poorly with disease severity, and symptoms are typically not altered significantly by currently available therapies (i.e. UDCA in PBC patients) (59, 60). The peripheral signaling pathways that link the diseased liver to the brain in cholestatic patients, to give rise to these disease-associated sickness behaviors, are not well understood (29). Our demonstration that ADAM17 inhibition attenuates sickness behavior development in our model highlights a potential novel therapeutic avenue for treating these symptoms in cholestatic patients, which are currently very challenging to manage in the clinic.

Given our current findings that ADAM17 plays an important role in regulating liver injury and sickness behavior development in BDL mice, we next delineated changes in hepatic ADAM17 expression in the setting of two important cholestatic liver diseases in patients; PBC and PSC. Indeed, similar to our observations in BDL mice, we found that both PBC and PSC were associated with an increase in hepatocyte ADAM17 protein expression which was most evident in hepatocytes identified as being closest to areas of active liver inflammation and parenchymal destruction. Given that ADAM17 has numerous targets important for regulating inflammation, increased ADAM17 expression in these areas likely contributes to a concerted change in the parenchymal and immune landscape within the local inflammatory milieu. Specifically, the ADAM17-mediated enhanced release of proinflammatory cytokines within the liver microenvironment, such as cleaving soluble TNFα from the cell surface of infiltrating or resident hepatic immune cell populations (44), likely significantly contributes to liver injury and has been implicated in the pathogenesis of both PBC and PSC (19, 21, 61, 62). The TNFα receptor TNFRI is richly expressed on the hepatocyte cell surface, and activation of membrane expressed TNFRI by TNFα can induce hepatocyte apoptosis (63, 64). Moreover, hepatocytes themselves may contribute to the inflammatory milieu in the liver environment. Others have shown using a conditional knockout of ADAM17 in hepatocytes can attenuate liver injury and systemic proinflammatory signaling in endotoxin treated mice (47). The broad role of ADAM17 inhibition on liver injury can also be seen in studies linking ADAM17 activity and hepatic stellate cell signaling in the setting of liver fibrosis in NASH (65) and ADAM17 in overall NAFLD development (66). However, targeting ADAM17 to produce an overall beneficial effect during inflammation may also depend on the context and cause of injury within the liver environment. Contrary to what we have shown here, others report increased ADAM17 activity being protective through its TNFRI shedding, which reduces overall TNFRI available to bind TNFα on the hepatocyte surface. These ADAM17-mediated protective effects were observed in experimental *in vitro* endotoxin-induced liver injury (67), and *in vivo* ischemia/reperfusion injury in rats (68). These differential findings from those in our study may be due to limitations of the *in vitro* assays, animal models utilized, and potentially mechanistic differences in underlying drivers of liver injury; endotoxin and ischemia primarily affecting hepatic vasculature in contrast to BDL-related liver injury that is driven by cholestasis and hepatic retention of mediators normally secreted in bile. Given this, the proinflammatory role of ADAM17 may be unique to certain etiologies of liver injury and warrants further investigation. Future studies evaluating the role of ADAM17 in other forms of liver disease including viral infection, autoimmune hepatitis, and drug induced liver injury may shed more light into this context dependent role of ADAM17 in liver health.

Cholangiocytes are target cells in both PBC and PSC, with bile duct damage and proliferation commonly observed in both of these human diseases. Similarly, bile duct proliferation has also been described in murine BDL models (69). We identified increased ADAM17 protein expression in cholangiocytes in liver biopsies obtained from PBC and PSC patients, compared to normal liver tissue. Moreover, we found similar enhanced cholangiocyte ADAM17 expression in BDL mice. Others have observed that cholangiocytes can regulate inflammatory responses within the liver (70, 71). One such mechanism includes signaling through the epidermal growth factor receptor (EGFR) expressed on cholangiocytes, which can be activated by several ligands, including transforming growth factor α (TGFα). The activation of EGFR in this fashion is important for stimulating cholangiocyte proliferation and repair processes. ADAM17-mediated secretion of TGFα may therefore have important implications for the regulation of bile duct inflammation, proliferation, and destruction in cholestatic diseases; a suggestion that is supported by our current findings of reduced bile duct proliferation in DPC 333-treated BDL mice. Interestingly, we also observed ADAM17 expressing immune cells infiltrating bile duct epithelium in both PBC and PSC patients, a process that may further alter cholangiocyte function, signaling, and responses to cholestasis. Further studies are required to better characterize the role of ADAM17 within the liver microenvironment and inflammatory milieu as it pertains to cholangiocyte response, bile duct injury, TGFα, EGFR signaling and overall cholestasis.

Expression of ADAM17 on the immune cell surface may also be an important regulator of tissue immune responses within the liver through the release of cell surface proteins involved in inflammatory processes, including cytokines (TNFα), their receptors (TNFRI, TNFRII, IL-6Rα), and adhesion molecules (L-selectin, VCAM-1) (5, 7, 10–12). We found that immune cell infiltrates in PBC and PSC patient livers were predominately T cells; an observation consistent with previous reports (72, 73). Moreover, within these hepatic T cell inflammatory infiltrates in PBC/PSC patients we readily identified both ADAM17^+^ CD4 and CD8 T cells. Consistent with our current findings, ADAM17 expression has previously been identified at low levels on human CD4 T cell surface membranes, and are transiently mobilized to the cell surface membrane during cell activation, ultimately regulating the release of soluble IL-6Rα (74) and enhancing FasL processing (75, 76), both effects having the potential to drive tissue injury and inflammation. In contrast, others have reported effector CD8 T cell activation during influenza infection resulting in ADAM17-mediated TNFRII cleavage and release from the cell surface membrane. Soluble TNFRII released in this manner critically regulates influenza-related pulmonary inflammation by binding to and inhibiting the proinflammatory effects of soluble TNFα released within the lung (77). In our study, we observed an amelioration of both BDL-associated liver injury and sickness behavior development with DPC 333-mediated ADAM17 inhibition, suggesting that in the setting of cholestatic liver injury, ADAM17 plays a more proinflammatory role. In addition to lymphocytes, ADAM17 is also expressed on the cell surface of macrophages and monocytes and is an important regulator of proinflammatory cytokine and cytokine receptor release from these cells during inflammation. For example, ADAM17 is the major ‘sheddase’ for both TNFα and TNFRI/TNFRII from activated macrophages (78). In liver biopsies from PBC and PSC patients, we readily identified ADAM17^+^ CD68^+^ macrophages/monocytes. Similar to our findings in the livers of PBC/PSC patients, we also identified significant increases in both ADAM17^+^ T cells and monocytes within the livers of BDL mice using flow cytometry. The relative contribution made by hepatic infiltration of ADAM17^+^ immune cells in the regulation of the overall inflammatory response within the liver environment remains unclear. A potential role for both myeloid- and lymphocyte-associated ADAM17 regulation of liver damage have been previously reported. Specifically, deletion of ADAM17 in myeloid cells completely prevented carbon tetrachloride-induced acute hepatitis, and attenuated endotoxin-induced elevations in circulating TNFα levels (47). The duality of ADAM17 to seemingly regulate both pro- and anti-inflammatory outcomes reflects the importance of understanding the context in which ADAM17 functions. Heterogeneity in terms of the site of end organ injury, agent/model of organ injury, and the subsequent predominant immune cell compartment(s) involved in driving injury, may all contribute to differing reports of the role ADAM17 plays in various inflammatory conditions. Our study identifies increased ADAM17 expression and activity as a proinflammatory driver of liver injury and sickness behavior development in a murine model of cholestasis, and also shows that ADAM17 expression is similarly enhanced in the liver of PBC and PSC patients in close proximity to areas of portal inflammatory cell infiltration, inflammation and hepatocyte injury. Therefore, our findings are consistent with ADAM17 playing a predominant proinflammatory role in the cholestatic liver environment.

There are limitations in our study that warrant further discussion. The BDL model does lead to measurable fibrosis on day 10 post surgery (79); however, chronic cholestasis and chronic fibrosis do not develop within this limited time period. In our hands, mice kept over the 10 day period post BDL surgery can lose more than 20% of their body weight, which meets our defined humane endpoint, making the study of chronic cholestasis difficult with the BDL model. Despite these model limitations, the BDL model have reproducable hepatic inflammatory environments representative of cholestasis (i.e., cholangiocyte injury, ductular reaction, portal inflammation and acute fibrosis), and associated sickness behaviours. Future studies could investigate the role of ADAM17 regulation in the context of chronic cholestasis in *Mdr2* or *Atp8b1* (80, 81) mutant mice. Evaluating ADAM17 in these models would better capture the role of ADAM17 inhibition in the context of longer term cholestatic liver injury. These genetic strains themselves have limitations however, as these specific mutations are not universally present in PBC/PSC patients and are more likely representative of genetic cholestatic liver disease in humans such as progressive familial intrahepatic cholestasis. Furthermore, we utilized only one selective ADAM17 inhibitor (i.e., DPC 333) in this study. The advent of oral small molecule therapy is altering the therapeutic landscape of chronic inflammatory conditions (82). TMI-005 is an oral small molecule inhibitor of ADAM17 and has been studied in various inflammatory conditions including Rheumatoid Arthritis (83). The use of TMI-005 could be considered in future studies to broaden the generalizability of our findings of ADAM17 inhibition in cholestasis. However, it is important to note that TMI-005 has inhibitory activity that extends beyond ADAM17 to include Matrix Metalloproteinase (MMP) 1 and 13. Whether concurrent inhibition of MMP-1 and MMP-13 can act synergistically with ADAM17 remains uncharacterized in cholestatic liver disease. Overall, our study highlights the role and potential for targeting ADAM17 in cholestatic liver injury.

In summary, hepatic ADAM17 expression and activity are enhanced in the setting of the cholestatic liver injury, and ADAM17 inhibition significantly attenuates liver damage and sickness behavior development in cholestatic mice. Moreover, enhanced ADAM17 expression in PBC/PSC patient liver biopsy samples was associated with hepatocytes, cholangiocytes, and was most evident in inflamed portal areas and in areas of parenchymal injury and immune cell infiltration. As such, ADAM17 inhibition may represent a novel therapeutic approach for patients with PBC and PSC, treating both disease-associated liver damage and adverse behavioral symptoms. Moreover, given the current lack of effective therapeutic options for treating PSC, inhibition of ADAM17 activity may represent a new approach for the treatment of this progressive and devastating disease.

## Data Availability Statement

The raw data supporting the conclusions of this article will be made available by the authors, without undue reservation.

## Ethics Statement

The studies involving human participants were reviewed and approved by Conjoint Health Research Ethics Board Research Services Office University of Calgary. The patients/participants provided their written informed consent to participate in this study. The animal study was reviewed and approved by Health Sciences Animal Care Committee (HSACC) University of Calgary.

## Author Contributions

HN conceived, designed, and performed the experiments. LS, CD’M, TL, and WA planned and performed the experiments. SU scored human and animal liver samples in a blinded fashion. HN and LS drafted and edited the manuscript. All authors provided critical feedback to shape the research, analysis, and final version of the manuscript.

## Funding

Funding from Cal Wenzel Family Foundation Chair in Hepatology.

## Conflict of Interest

The authors declare that the research was conducted in the absence of any commercial or financial relationships that could be construed as a potential conflict of interest.

## Publisher’s Note

All claims expressed in this article are solely those of the authors and do not necessarily represent those of their affiliated organizations, or those of the publisher, the editors and the reviewers. Any product that may be evaluated in this article, or claim that may be made by its manufacturer, is not guaranteed or endorsed by the publisher.
